# Validity of multiplex biomarker model of 6 genes for the differential diagnosis of thyroid nodules

**DOI:** 10.1186/1756-6614-4-11

**Published:** 2011-06-27

**Authors:** Kristine Ducena, Arturs Ābols, Janis Vilmanis, Zenons Narbuts, Juris Tārs, Diana Andrējeva, Aija Linē, Valdis Pīrāgs

**Affiliations:** 1 Faculty of Medicine, University of Latvia, Raina Bulvaris 19, Riga, LV1586, Latvia; 2 Department of Molecular Biology, Latvian Biomedical Research and Study Centre, Ratsupites 1,Riga, LV1067, Latvia; 3 Surgery Clinics, Pauls Stradins Clinical University Hospital, Pilsonu 13, Riga, LV1002, Latvia; 4 Oncology department, Riga Eastern Clinical University Hospital, Hipokrata 2, Riga, LV1038, Latvia

## Abstract

**Background:**

Currently the cytological examination of fine needle aspiration (FNA) biopsies is the standard technique for the pre-operative differential diagnosis of thyroid nodules. However, the results may be non-informative in ~20% of cases due to an inadequate sampling and the lack of highly specific, measurable cytological criteria, therefore ancillary biomarkers that could aid in these cases are clearly needed. The aim of our study was to evaluate the mRNA expression levels of 8 candidate marker genes as the diagnostic biomarkers for the discrimination of benign and malignant thyroid nodules and to find a combination of biomarkers with the highest diagnostic value.

**Materials and methods:**

mRNA expression levels of eight candidate marker genes - *BIRC5, CCND1, CDH1, CITED1, DPP4, LGALS3, MET *and *TFF3 *was measured by real-time RT-PCR in paired nodular and surrounding normal thyroid tissue specimens of 105 consecutive patients undergoing thyroid surgery and compared between different types of thyroid lesions.

**Results:**

Significant differences in the mRNA expression levels between the normal and malignant thyroid tissues and between benign and malignant nodules were found for *BIRC5, CCND1, CITED1, DPP4, LGALS3, MET *and *TFF3*, but not *CDH1*. On a single gene basis, relative quantity (RQ) of *LGALS3 *had the highest diagnostic value for the discrimination of malignant and benign thyroid nodules (AUC = 0.832, P < 0.0001 and 90.9% sensitivity and 65.6% specificity at the optimal cut-off on ROC curve). The only two-marker set that outperformed *LGALS3 *was RQ sum of *LGALS3 *and *BIRC5 *(AUC = 0.841, P < 0.0001). An application of multivariate logistic regression analysis resulted in the generation of a multiplex biomarker model based on *LGALS3, BIRC5, TFF3, CCND1, MET *and *CITED1 *that had considerably higher specificity than a single marker or two marker gene-based models (AUC = 0.895, P < 0.0001, 70.5% sensitivity and 93.4% specificity).

**Conclusions:**

This study confirmed that mRNA expression levels of 7 out of 8 candidate genes analysed have a diagnostic value for the distinction of benign and malignant thyroid nodules. The multiplex biomarker model based on 6 genes outperformed a single marker or two marker-based models and warrants feasibility studies on FNA biopsies and the validation in a larger cohort of patients.

## Background

Thyroid nodules are a very common clinical finding - the prevalence of palpable nodules ranges from 3 to 7% in the general population but can be as high as 50% based on ultrasonography or autopsy data [1]. Although only less than 5% of the palpable nodules are malignant lesions, thyroid cancers are the most common malignancy of endocrine organs. According to European Cancer Observatory data age-standardised incidence rate was 3.1 cases and 8.8 cases per 100 000 in men and women, respectively in Europe in 2008 and its incidence rates have steadily increased over the recent decades [2,3]. More than 95% of malignant lesions are derived from thyroid follicular cells and are divided into papillary and follicular carcinomas that differ mainly in the mode of metastatic spread (lymphatic and haematogenous spread, respectively) yet both are relatively indolent tumours with 5-year survival rates > 90%, and undifferentiated or anaplastic carcinoma that is a highly aggressive and lethal cancer with 5-year survival rate below 1-17% [4]. A minority of thyroid carcinomas are derived from parafollicular cells (or C-cells) and are referred to as medullary carcinomas with 5-year survival rate of ~80% [4]. Benign nodules include hyperplasic follicular adenomas, multinodular goiter, thyroiditis and benign cysts [1].

The differential diagnosis of nodular thyroid disease is based on cytological examination of fine needle aspiration (FNA) biopsies, however the results may be non-informative in ~20% of cases due to an inadequate sampling and the lack of highly specific, measurable cytological criteria [4,5]. Furthermore, the principal diagnostic feature for follicular thyroid carcinomas (FTC) is capsular or vascular invasion and therefore it can not be reliably distinguished from follicular adenomas by analysis of FNA smears and the definite diagnosis relies on the histological examination of the postoperative surgical specimens [6]. Hence molecular biomarkers of malignancy that could reliably discriminate between malignant and benign nodules in the grey zone of thyroid FNA and classify tumours into the histological subtypes are clearly needed.

Gene expression profiling using cDNA microarrays or serial analysis of gene expression (SAGE) has revealed several hundreds of genes that are differentially expressed between malignant and benign thyroid nodules [7-9]. A number of them, including *LGALS3, MET *etc have been shown to be functionally involved in the carcinogenic process, have been validated by qRT-PCR and confirmed at protein level by immunohistochemistry [10-12]. However, several studies have demonstrated that none of these genes individually has sufficient sensitivity and specificity to be exploited as an independent diagnostic biomarker [13,14] while using very large panels of genes may not be practical. Therefore the definition of minimal set of marker genes that allows the correct classification of nodules is required in order to develop a clinically applicable biomarker assay. In the current study, we evaluated the diagnostic value of 8 candidate marker genes - *BIRC5, CCND1, CDH1, CITED1, DPP4, LGALS3, MET *and *TFF3 *by analysing their mRNA expression levels in nodular and adjacent relatively normal thyroid tissue specimens of 105 consecutive patients with thyroid nodules who underwent thyroidectomy, and developed a multiplex biomarker model for the classification of the nodules.

## Methods

### Patients and tissue specimens

Paired specimens of thyroid nodules and surrounding normal tissues were collected from 105 consecutive patients undergoing total or partial thyroidectomy at the Latvian Centre of Oncology and Pauls Stradi	ņš Clinical University Hospital during the period 2009-2010. Tissue samples were macroscopically dissected by histopathologist during the surgery and stored in RNALater^® ^(Applied Biosystems, USA) at -20°C until processing. Tissue sections were evaluated by experienced pathologist and the diagnosis was established according to standard histopathological criteria. Sixty one of the nodules were diagnosed as follicular adenomas (FA), 33 as papillary thyroid carcinomas (PTC), 5 as medullar thyroid carcinomas (MTC), 3 as anaplastic thyroid carcinomas (ATC) and 3 as follicular thyroid carcinomas (FTC). Selected clinical-pathological data of the patient groups are provided in Table 1. The specimens were collected after the patients' informed consent was obtained in accordance with the regulations of Ethics Committee of the University of Latvia Institute of Experimental and Clinical Medicine.

**Table 1:** Clinical and pathological characteristics of the patient groups

Characteristics	Thyroid cancer n = 44	Benign nodule n = 61
**Gender**		

Male	9	4
Female	35	57

**Age at diagnosis**		

Mean ± SD	55 ± 17	54 ± 15
Median (range)	55 (24-83)	57 (25-83)

**Family history of cancer**	37	49

**TSH level μIU/mL**		

Mean ± SD	2,31 ± 1,41	0,87 ± 0,74
Median (range)	1,32 (1,1-4,34)	0,75 (0,004-2,43)

**US criteria**		

Hipoechogenic nodule	9	14
No Halozones	0	1
Irregular frontier (non-homogenic)	6	14
Microcalcinates	4	9
Nodules > 3 cm	5	19
Central vascularization	3	3
Retrosternal	1	7
Swollen lymph nodes	2	0
No data	18	8

**Histopathology**		

PTC	33	
FTC	3	
MTC	5	
ATC	3	
FA		61

### RNA extraction and cDNA synthesis

Tissue specimens were homogenised using the FastPrep-24 instrument and Lysing Matrix D (MP Biomedicals, USA) in 1 ml of Lysis solution^® ^(Ambion, USA) followed by the extraction of total RNA using MirVana^® ^(Ambion, USA) according to manufacturer's instructions. RNA extracted from tissue material was treated with DNAse prior to cDNA synthesis (Ambion, USA) and quantified by NanoDrop ND-100 spectrophotometer. cDNA was synthesized by random hexamer priming from 4 μg of total RNA by using High-Capacity cDNA Reverse Transcription Kit (Applied Biosystems, USA) according to manufacturer's instructions.

### Quantitative RT-PCR

Quantitative RT-PCR (qPCR) reactions were performed using 2 μl of 1:10 diluted cDNA reaction mixtures, ABSolute Blue™ SYBR green Low ROX (Thermo Scientific, USA) on ABI7500 sequence detection system (Applied Biosystems, USA). Appropriate primer concentrations were established by cDNA 4 log serial dilution curves to ensure amplification efficiency over 95% and the specificity of the amplification products were verified by the melting curve analysis. Sequences of primers used in this study are provided in Table 2. qPCR conditions were as follows: hold for 15 minutes at 95°C, follow by two step PCR for 40 cycles of 95°C for 15 seconds, followed by 60°C for 1 minute. All reactions were performed in duplicates. To choose the appropriate reference genes, a set of seven candidate reference genes (*PGK1, PLA2, TUBA3, ACTB, GAPDH, TBP, POLR2A*) was tested for their mRNA expression stability in 10 thyroid tissue samples from 5 patients (2 PTC, 3 FA, 5 normal) and the two most stable genes (*POLR2A and PGK1*) were determined by using the open-access software GeNorm Win3.4 (http://medgen.ugent.be/~jvdesomp/genorm). Normalisation factor (NF) for each cDNA sample was then calculated by using GeNorm Win3.4 based on *PGK1 *and *POLR2A *expression level. Pooled cDNA from 10 thyroid tissue samples was used as a reference sample and included in each experiment to allow the comparison across multiple plates. The expression level of each gene was determined relative to its expression in the sample with the highest expression (lowest Ct value). The analysis of relative gene expression (RQ) data was performed using the 2^-ΔCT ^method and to eliminate variations of cDNA quantity and quality the data were normalised by using NF for each sample (RQ = (2^-ΔCT^)/NF).

**Table 2:** Primers used for qPCR

Name of the gene	Sequence (5'- > 3')	Size of PCR product (bp)	**GenBank accession no**.
**Candidate genes**			

LGALS3 F	CTGATTGTGCCTTATAACCTGC	100	NM_002306.3
LGALS3 R	AAGCAATTCTGTTTGCATTGG		

TFF3 F	GTACGTGGGCCTGTCTGC	121	NM_003226.3
TFF3 R	GATCCTGGAGTCAAAGCAGC		

DPP4 F	TGATGCTACAGCTGACAGTCG	164	NM_001935.3
DPP4 R	CTGAGCTGTTTCCATATTCAGC		

CITED1 F	GCTCTGAAATGCCAACAACG	174	NM_001144886.1
CITED1 R	TGGTTCCATTTGAGGCTACC		

MET F	TCTGCCTGCAATCTACAAGG	153	NM_001127500.1
MET R	AAGGTGCAGCTCTCATTTCC		

CDH1 F	AGAAACAGGATGGCTGAAGG	199	NM_004360.3
CDH1 R	AGCACCTTCCATGACAGACC		

CCND1 F	TGGTGAACAAGCTCAAGTGG	280	NM_053056.2
CCND1 R	ATCACTCTGGAGAGGAAGCG		

BIRC5 F	CAGCCCTTTCTCAAGGACC	152	NM_001168.2
BIRC5 R	AAGCAGAAGAAACACTGGGC		

**Hausekeeping genes**			

PGK1 F	CTTAAGGTGCTCAACAACATGG	119	NM_000291.3
PGK1 R	ACAGGCAAGGTAATCTTCACAC		

POLR2A F	GGGTCATCTTCCCAACTGGAG	164	NM_000937.4
POLR2A R	CACCAGCTTCTTGCTCAATTCC		

F - forward primer, R- reverse primer

### Statistical analysis

A non-parametric Mann-Whitney *U *test was used to compare the RQ values of each candidate gene between two independent groups of samples (benign vs malignant nodules; malignant nodules vs all normal tissue specimens). The statistical significance was set at p < 0.05. The receiver operating characteristic (ROC) curve was constructed and the area under the curve (AUC) was calculated to evaluate the diagnostic performance of each marker gene and biomarker model. Youden index (J) was used to define cut-off points on the ROC curves with the maximal sum of sensitivity and specificity. Multivariate logistic regression was used to examine associations between thyroid nodule histopathological status and the gene expression data as described by Laxman B et al [15]. The initial multivariate logistic regression model included all individual genes and all combinations. Stepwise backward elimination based likelihood ratio test was used to drop out insignificant terms from the initial model. The predicted probability for each sample was calculated and used as input to generate ROC curve. Leave one-out cross validation (LOOCV) as described earlier was used to validate biomarkers and combinations of them to eliminate overestimated values [15]. The statistical analyses were performed with SPSS 17.0 (SPSS, USA), Genex (Multid, Sweden) and GraphPadPrism 5 (GraphPad, USA).

## Results and discussion

### Selection of candidate marker genes

The selection of candidate marker genes was based on the functional involvement in various pathways contributing to the acquisition of malignant phenotype and/or previously reported differential expression in the malignant and benign thyroid nodules. Cyclin D1 encoded by *CCND1 *is a crucial cell cycle regulator frequently upregulated at protein level in PTC [16]. *CITED1 *encodes a cell cycle-dependent transcriptional cofactor involved in TGF-beta and Bmp signalling that may coordinate cellular differentiation and survival signals [17,18] and has been found to be overexpressed in PTC by expression profiling using cDNA microarrays [19], later confirmed as useful marker for FTC [6] and validated at protein level by IHC [20]. Loss of expression or function of E-cadherin - a cell-cell adhesion glycoprotein encoded by *CDH1*, has been demonstrated to contribute to the progression of various cancers, including poorly differentiated thyroid cancers by increasing proliferation, invasion and metastasis [21,22]. Overexpression of dipeptidyl-peptidase 4 encoded by *DPP4 *in PTC has been demonstrated at protein level by several groups [23-25], however the mechanism how it may contribute to the development or progression of thyroid malignancies remains unknown. Survivin, an inhibitor of apoptosis encoded by *BIRC5*, has been shown to be overexpressed in a variety of cancers, including thyroid cancer, where it contributes to uncontrolled cancer cell growth and drug resistance [26,27]. Increased expression at the mRNA and protein level of the receptor for hepatocyte growth factor encoded by *MET *has been frequently observed in PTC, follicular variant of PTC and at lesser degree in FTC where it promotes tumour progression by facilitating cell proliferation, survival, migration, invasion, and metastasis [28-30]. However, to our knowledge, so far the diagnostic value of *CDH1, DPP4, BIRC5 *and *MET *mRNA levels has not been established. Galectin 3 encoded by *LGALS3 *plays an important role in cell-to-cell adhesion, cell-to-matrix interactions and the regulation of apoptosis and proliferation, and its overexpression correlates with thyroid cancer aggressiveness and metastasis [31,32]. Although *TFF3 *mRNA has been shown to be overexpressed in several solid cancers, such as hepatocellular carcinoma, colon and prostate cancer, and suggested to contribute the malignant behaviour and metastasis [33-36], SAGE analysis of thyroid follicular adenomas and carcinomas demonstrated that it is downregulated in FTC [37] and later this finding was confirmed in PTC and ATC by other researchers [14,38], thus validating *TFF3 *downregulation as a universal marker of cancers derived from thyroid follicular cells. It encodes a small secreted protein - trefoil factor 3, which is abundantly expressed at mucosal surfaces and promotes regeneration and repair. Interestingly, recent reports demonstrate that both galectin-3 and TFF3 are involved in ciliogenesis, epithelial cell differentiation and polarity [39,40], thus suggesting a yet unexplored role of the deregulation of these processes in the development of thyroid cancer.

### mRNA expression analysis and the diagnostic performance of individual marker genes

**Figure 1:**
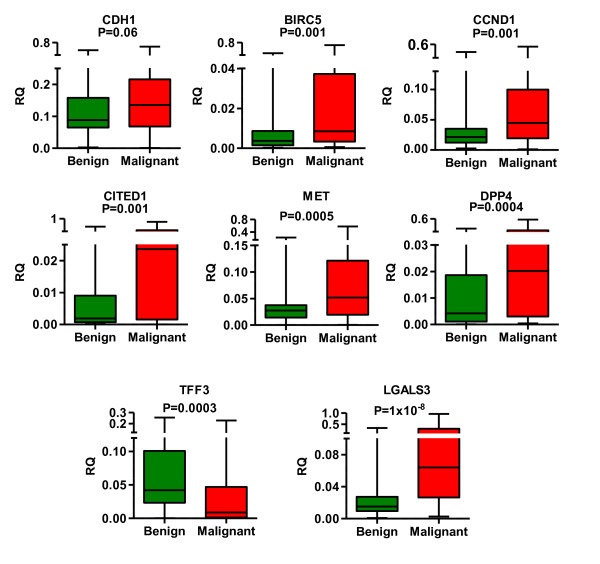
**Median mRNA expression levels of the candidate marker genes in groups of 61 benign and 44 malignant thyroid nodules represented by box plots**. The relative quantity (RQ) of each marker gene in each sample was calculated using 2^-ΔCT ^method and normalised by using normalisation factor obtained by analysis of the two most stable reference genes (RQ = (2^-ΔCT^)/NF). Boxes represent the 25^th ^and 75^th ^percentiles; whiskers represent the minimum and maximum values. Statistical significance was calculated using Mann-Whitney *U *test.

**Figure 2:**
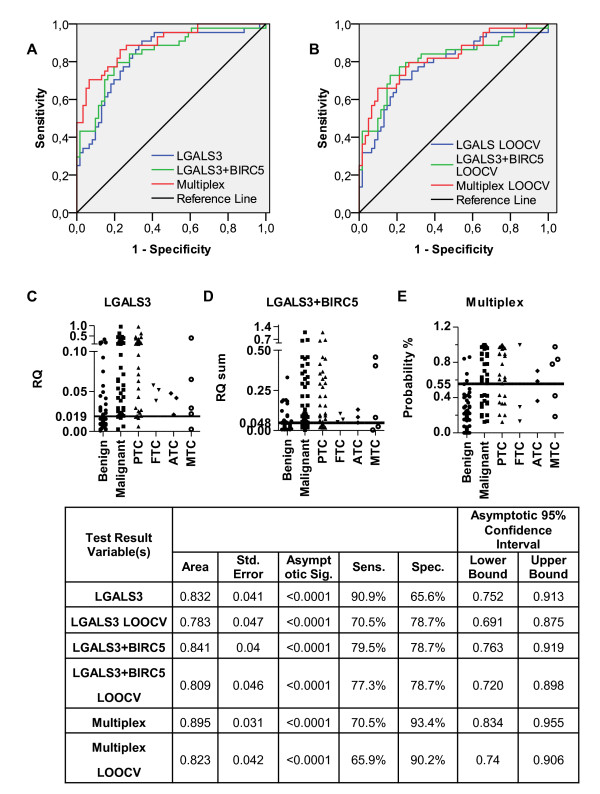
**The diagnostic performance of the best individual marker, two-marker set and multiplex biomarker model**. A and B represents ROC curves of the best individual marker, two-marker set and multiplex model and their LOOCV ROC curves, respectively. C, D, E - Scatter dot plot of LGALS3 RQ, LGALS3 and BIRC5 RQ sum and Multiplex model with the best cut off shows how many benign nodules, malignant nodules and thyroid cancer subtypes are misclassified. In table are the best individual marker, two-marker set and multiplex biomarker model and their LOOCV model ROC curve AUC data and sensitivity and specificity at the best cut-off point.

mRNA expression analysis of the selected marker genes by RT-qPCR revealed statistically significant differences both between the normal and malignant thyroid tissues and between benign and malignant nodules for all genes except *CDH1*, thus further supporting their role the development and/or progression of thyroid cancer. *LGALS3, DPP4, MET, CITED1, CCND1 *and *BIRC5 *were found to be significantly upregulated while *TFF3 *was downregulated in the malignant tissues (Table 3 and Figure 1). At first, all the genes were tested by ROC curve analysis as individual biomarkers, which demonstrated that *LGALS3 *have the highest value for discriminating malignant from benign nodules in our sample set (AUC = 0.832, P < 0.0001). The cut-off of *LGALS3 *expression level (RQ) that discriminates benign and malignant nodules with the highest accuracy (sensitivity 90.9%, specificity 65.6%) was determined to be 0.019. The sensitivity for the detection of various subtypes of thyroid cancers was mutually comparable (Figure 2C) - 29 out of 33 PTC, 4 of 5 MTC and all FTC and ATC specimens were classified correctly thus confirming that *LGALS3 *may serve as a universal diagnostic marker of the thyroid malignancies. However, 22 of 61 benign nodules were misclassified using this cut-off. As the ROC curve was constructed using all the samples, it may lead to overestimation of the AUC, therefore we next used LOOCV to validate it. The AUC for LOOCV *LGALS3 *model dropped to 0.783, nevertheless it still outperformed the other marker genes. Although previously the diagnostic utility of *LGALS3 *mRNA expression level has been questioned, our data show that the diagnostic accuracy of mRNA quantification is comparable to that reported in immunohistochemical studies [32]. The next marker genes with the highest diagnostic value in our sample set were *TFF3 *(AUC = 0.696, P = 0.001) and DPP4 (AUC = 0.695 and P = 0.001). The optimal RQ cut-off for *TFF3 *was determined to be 0.011 allowing the discrimination between benign and malignant nodules with sensitivity 54.6% and specificity 90.2%, while for *DPP4 *the cut-off was 0.027 with sensitivity 45.6% and specificity 88.5%.

**Table 3:** Relative expression values and the diagnostic performance of the marker genes

Gene, protein name	RQ values - Mean ± SEM			Mann-Whitney U test - p-values		ROC curve - AUC (Asimptotic significance)	
	
	Normal	Benign	Malignant	Normal vs Malignant	Benign vs Malignant	Normal vs Malignant	Benign vs Malignant
*LGALS3*, Galectin-3	0.068 ± 0.013	0.035 ± 0.007	0.248 ± 0.067	2.2 × 10^-5^	1 × 10^-8^	0.715 (< 0.0001)	0.832 (< 0.0001)

*TFF3*, trefoil factor 3	0.115 ± 0.011	0.066 ± 0.007	0.037 ± 0.008	4.1 × 10^-8^	0.0003	0.782 (< 0.0001)	0.696 (0.001)

*DPP4*, Dipeptidyl peptidase-4	0.011 ± 0.002	0.0146 ± 0.003	0.066 ± 0.016	0.0001	0.0004	0.693 (< 0.0001)	0.695 (0.001)

*MET*, hepatocyte growth factor receptor	0.048 ± 0.005	0.033 ± 0.004	0.110 ± 0.021	0.01	0.0005	0.610 (0.037)	0.689 (0.001)

*CITED1*, Cbp/p300-interacting transactivator 1	0.010 ± 0.002	0.021 ± 0.008	0.161 ± 0.048	0.001	0.001	0.654 (0.003)	0.67 (0.003)

*CCND1*, Cyclin D1	0.037 ± 0.004	0.036 ± 0.007	0.084 ± 0.017	0.002	0.001	0.647 (0.005)	0.67 (0.003)

*BIRC5*, Survivin	0.025 ± 0.011	0.009 ± 0.002	0.045 ± 0.018	0.0001	0.001	0.694 (< 0.0001)	0.668 (0.003)

*CDH1*,E-cadherin	0.171 ± 0.013	0.117 ± 0.009	0.158 ± 0.018	0.2	0.06	0.464 (0.497)	0.525 (0.697)

Interestingly, in 3 PTC and 2 MTC cases none or only one of the genes analysed were differentially expressed between cancerous and relatively normal tissues in specimens and they were consistently misclassified as benign nodules. These specimens are likely to represent a distinct molecular subtype of thyroid cancer, in which different molecular pathways predominate and therefore next these samples will be subjected to gene expression profiling using cDNA microarrays.

### Development of multiplex biomarker model

To determine if a combination of markers could outperform the single biomarkers we systematically searched for two-marker ratios or two-marker sums, and determined their diagnostic performance by ROC curve analysis. In total, 27 two-marker combinations were evaluated, however only one of them - *LGALS3 *and *BIRC5 *RQ sum showed higher AUC for discriminating benign and malignant nodules than the best individual marker gene (AUC = 0.841, P < 0.0001 and AUC = 0.809, P < 0.0001 for the LOOCV model) (Figure 2A,B). At the best cut-off, the sensitivity was 79.5% and specificity was 78.7%. As shown in Figure 2D, this two-gene combination could detect all FTC and ATC cases but failed to detect 7 of 33 PTCs (21%) and 2 of 5 MTCs (40%) thus suggesting it may have lower value for diagnosing MTC, however this finding should be verified in larger cohort of patients. So far, the best reported two-marker set is *TFF3/LGALS3 *ratio that has been shown to discriminate follicular carcinomas from follicular adenomas with 72.4% sensitivity and 83.3% specificity (or 80% and 91.5%, when the pathologically questionable cases were excluded) [41]. In our sample set it showed similar performance (AUC = 0.758, P < 0.0001, sensitivity 72.7%, specificity 85.3%). However, although it could improve the specificity, the overall accuracy of *TFF3/LGALS3 *ratio was lower than that of *LGALS3 *alone. Moreover, as *TFF3 *is downregulated in cancer cells, the development of assay that is based on the measurement of its expression level in FNA biopsies consisting of a mixture of different cell types is technically more challenging than the assay based on overexpressed genes, therefore *LGALS3 *and *BIRC5 *RQ sum seems to be more suitable for the clinical application.

Next, we applied a multivariate logistic regression analysis to define a multiple marker set that could improve the diagnostic performance over single markers or two-marker combinations. Similar approach has been previously successfully used by Laxman B et al to develop a multiplex biomarker model for the detection of prostate cancer [15]. This resulted in the multiplex model that included LGALS3 and BIRC5 RQ sum, TFF3 and LGALS3 RQ ratio, TFF3 and CCND1 RQ ratio, ratio between TFF3 RQ and MET, CITED1 RQ sum, ratio between TFF3 RQ and MET, BIRC5 RQ sum and ratio between TFF3 RQ and CCND1, BIRC5 RQ sum. Although the overall performance of the model (AUC = 0.895, P < 0.0001 and AUC = 0.823, P < 0.0001 for the LOOCV model) was only slightly improved over *LGALS3 *alone or *LGALS3 *and *BIRC5 *RQ sum, at the best cut-off, this model shows 70.5% sensitivity and 93.4% specificity and as shown in Figure 2A it has considerably higher specificity that is a clear requirement for the development of clinically applicable biomarker assay.

The future efforts will be focused on adding marker genes to the multiplex model that would enable detecting other molecular subtypes of thyroid cancer, testing the feasibility of the biomarker assay in FNA biopsies and validating the assay in a large multicenter clinical trial.

## Conclusions

mRNA expression analysis of 8 candidate marker genes - *BIRC5, CCND1, CDH1, CITED1, DPP4, LGALS3, MET *and *TFF3 *in paired nodular and relatively normal thyroid tissue specimens of 105 consecutive patients undergoing thyroid surgery demonstrated that all of them except *CDH1 *are differentially expressed between the normal and malignant thyroid tissues and between benign and malignant nodules, and *LGALS3 *had the highest diagnostic value for the discrimination of malignant and benign thyroid nodules on a single gene basis. An application of multivariate logistic regression analysis resulted in the generation of a multiplex biomarker model based on *LGALS3, BIRC5, TFF3, CCND1, MET *and *CITED1 *that outperformed a single marker or two marker gene-based models by increasing the specificity, which is a prerequisite for the development of clinically applicable biomarker assay. The next step will be to test the feasibility of this assay on FNA biopsies and to validate it in a larger cohort of patients.

## Competing interests

The authors declare that they have no competing interests.

## Authors' contributions

KD participated in the designing of the study, collecting of clinical data and drafting the manuscript. AA carried out the gene expression analysis, performed the statistical analysis and helped to draft the manuscript. JV, ZN and JT performed thyroid surgery and collected the clinical material. DA participated in the gene expression analysis. AL participated in the design of the study and drafted the manuscript. VP designed and coordinated the study and helped to draft the manuscript. All authors read and approved the final manuscript.
